# Dose escalation for stereotactic arrhythmia radioablation of recurrent ventricular tachyarrhythmia - a phase II clinical trial

**DOI:** 10.1186/s13014-023-02361-x

**Published:** 2023-11-08

**Authors:** Boldizsar Kovacs, Michael Mayinger, Stefanie Ehrbar, Debra Fesslmeier, Maiwand Ahmadsei, Lorraine Sazgary, Robert Manka, Hatem Alkadhi, Frank Ruschitzka, Firat Duru, Alexandros Papachristofilou, Christian Sticherling, Slawomir Blamek, Krzysztof S. Gołba, Matthias Guckenberger, Ardan M. Saguner, Nicolaus Andratschke

**Affiliations:** 1 Department of Cardiology, University Heart Center, University Hospital Zurich, Zurich, Switzerland; 2 Division of Cardiology, Department of Internal Medicine, University of Michigan, Ann Arbor, USA; 3 Center for Translational and Experimental Cardiology (CTEC), University of Zurich, Zurich, Switzerland; 4 Department of Radiation Oncology, University Hospital Zurich, Zurich, Switzerland; 5 Diagnostic and Interventional Radiology, University Hospital Zurich, Zurich, Switzerland; 6 Center for Integrative Human Physiology, University Zurich, Zurich, Switzerland; 7 Department of Radiation Oncology, University Hospital Basel, Basel, Switzerland; 8 Department of Cardiology, University Hospital Basel, Basel, Switzerland; 9 Department of Radiotherapy, Maria Skłodowska-Curie National Research Institute of Oncology, Gliwice, Poland; 10 Department of Electrocardiology, Upper Silesian Heart Center, Medical University of Silesia, Katowice, Poland; 11 Department of Electrocardiology and Heart Failure, Medical University of Silesia, Katowice, Poland

**Keywords:** Stereotactic Arrhythmia Radioablation, Stereotactic body Radiotherapy, Ventricular tachycardia, Ventricular arrhythmia, Study protocol

## Abstract

**Background:**

Stereotactic arrhythmia radioablation (STAR) is delivered with a planning target volume (PTV) prescription dose of 25 Gy, mostly to the surrounding 75–85% isodose line. This means that the average and maximum dose received by the target is less than 35 Gy, which is the minimum threshold required to create a homogenous transmural fibrosis. Similar to catheter ablation, the primary objective of STAR should be transmural fibrosis to prevent heterogenous intracardiac conduction velocities and the occurrence of sustained ventricular arrhythmias (sVA) caused by reentry. We hypothesize that the current dose prescription used in STAR is inadequate for the long-term prevention of sVA and that a significant increase in dose is necessary to induce transmural scar formation.

**Objective:**

A single arm, multi-center, phase II, dose escalation prospective clinical trial employing the i3 + 3 design is being conducted to examine the safety of a radiation dose-escalation strategy aimed at inducing transmural scar formation. The ultimate objective of this trial is to decrease the likelihood of sVA recurrence in patients at risk.

**Methods:**

Patients with ischemic or non-ischemic cardiomyopathy and recurrent sVA, with an ICD and history of ≥ 1 catheter ablation for sVA will be included. This is a prospective, multicenter, one-arm, dose-escalation trial utilizing the i3 + 3 design, a modified 3 + 3 specifically created to overcome limitations in traditional dose-finding studies. A total of 15 patients will be recruited. The trial aims to escalate the ITV dose from 27.0 Gy to an ITV prescription dose-equivalent level of maximum 35.1 Gy by keeping the PTV prescription dose constant at 25 Gy while increasing the dose to the target (i.e. the VT substrate without PTV margin) by step-wise reduction of the prescribing isodose line (85% down to 65%). The primary outcome of this trial is safety measured by registered radiation associated adverse events (AE) up to 90 days after study intervention including radiation associated serious adverse events graded as at least 4 or 5 according to CTCAE v5, radiation pneumonitis or pericarditis requiring hospitalization and decrease in LVEF ≥ 10% as assessed by echocardiography or cardiac MRI at 90 days after STAR. The sample size was determined assuming an acceptable primary outcome event rate of 20%. Secondary outcomes include sVA burden at 6 months after STAR, time to first sVA recurrence, reduction in appropriate ICD therapies, the need for escalation of antiarrhythmic drugs, non-radiation associated safety and patient reported outcome measures such as SF-36 and EQ5D.

**Discussion:**

DEFT-STAR is an innovative prospective phase II trial that aims to evaluate the optimal radiation dose for STAR in patients with therapy-refractory sVA. The trial has obtained IRB approval and focuses on determining the safe and effective radiation dose to be employed in the STAR procedure.

**Trial registration:**

NCT05594368.

## Background

Cardiovascular diseases account for 32% of deaths globally and 45% of deaths in Europe [1, 2]. Sudden cardiac death (SCD) caused by sustained ventricular tachycardia and fibrillation (VT/VF) account for approximately 50% of these death [3]. The most common causes for VT/VF are ischemic or non-ischemic (i.e. dilated, hypertrophic, etc.) cardiomyopathies (ICM, NIMC) that are associated with myocardial scar formation facilitating the development of arrhythmias.

While implantable cardioverter defibrillators (ICD) reduce the risk SCD due to VT/VF, they do not prevent their occurrence. Therefore, VT/VF remain major cause of morbidity and mortality as well as reduced quality of life in patients with ischemic or non-ischemic cardiomyopathies. Certain antiarrhythmic drugs (in particular amiodarone) and catheter ablation (CA) reduce the rate of VT/VF and SCD [3, 4]. But although CA is efficacious in suppressing sustained monomorphic VT (MMVT) a total recurrence rate of 33.2% was reported in a recent meta-analysis including all randomized controlled trials comparing CA with conservative treatment [5]. Difficulty in reaching arrhythmogenic substrate (intramural or epicardial), large scars and other procedural factors may lead to VT recurrence after an intervention. Additionally, CA can be associated with major complications in up to 9.5% [6].

More recently, stereotactic arrhythmia radioablation (STAR) has been introduced as a non-invasive treatment for refractory sustained MMVT with the delivery of a single fraction high-dose radiotherapy [7–9]. This treatment aims to deliver focal treatment to the arrhythmogenic substrate to ultimately induce transmural fibrosis, similar to the goal of CA.

While initial trials reported a high immediate success rate, a systematic review of the available clinical data found frequent, sustained ventricular arrhythmia (sVA) recurrences after the index procedure in up to 75% of treated patients within the first year. The commonly employed prescription dose has been 25 Gy either homogeneously or inhomogeneously to the 70–85% isodose line and was shown to maintain a high degree of safety [10]. However, from animal experiments, it is evident that such a dose will not be able to induce transmural fibrosis within the myocardium, which is thought to be necessary for long-term success of VT ablation [11]. A probable explanation for these late recurrences may be the lack of such a transmural fibrosis development in the irradiated myocardium. This would be necessary to homogenize the scar to prevent the development of reentry circuits, the cause of MMVT [12]. Indeed, a mechanism alternative to scar homogenization by which STAR may prevent recurrent sVA has recently been proposed [13].

To date, all studies have focused on initial safety and have not yet explored a possible dose-effect relationship for developing transmural fibrosis. In human trials, to date a maximum dose of 25 Gy was prescribed to the target volume, with inhomogeneity allowed in some instances to amount to a maximum point dose of 33 Gy, which is below the anticipated threshold of 35 Gy for transmural scar formation [10, 14]. Pre-clinical trials have yielded similar findings [15]. Increasing the dose to the cardiac target to induce transmural scar formation is a promising approach to increase the long-term efficacy of STAR.

This study hypothesizes that the underlying mechanism of successful VT treatment is the induction of a homogeneous scar formation within the critical arrhythmic substrate for which a higher radiation dose than the currently applied 25 Gy prescription dose will be necessary. Therefore dose-escalation will lead to a significantly reduced long-term VT recurrence rate compared to the currently applied single dose of 25 Gy. This phase II study aims to identify the optimal dose - i.e. minimal efficacious dose required for successful STAR treatment, while not jeopardizing treatment safety.

## Methods

### Study aim

This study aims to demonstrate the safety of escalating the usually employed dose from 25 Gy prescribed to the 65–90% target volume encompassing isodose to a maximum of 32.5 Gy prescribed to the 65–90% with the ultimate goal of decreasing long-term sVA recurrence rates through homogeneous scar formation.

### Study design

DEFT-STAR is an ongoing single arm, multi-center, phase II, dose escalation trial employing the i3 + 3 design. The design has specifically been created for dose finding studies in radiation oncology. This design has been found to be more robust with regard to statistical operating characteristics than the classical 3 + 3 design [16]. After informed consent is obtained from participants, they will be assigned to a radiation dose group according to the dose escalation simulation.

### Sample size calculation

**Fig. 1:**
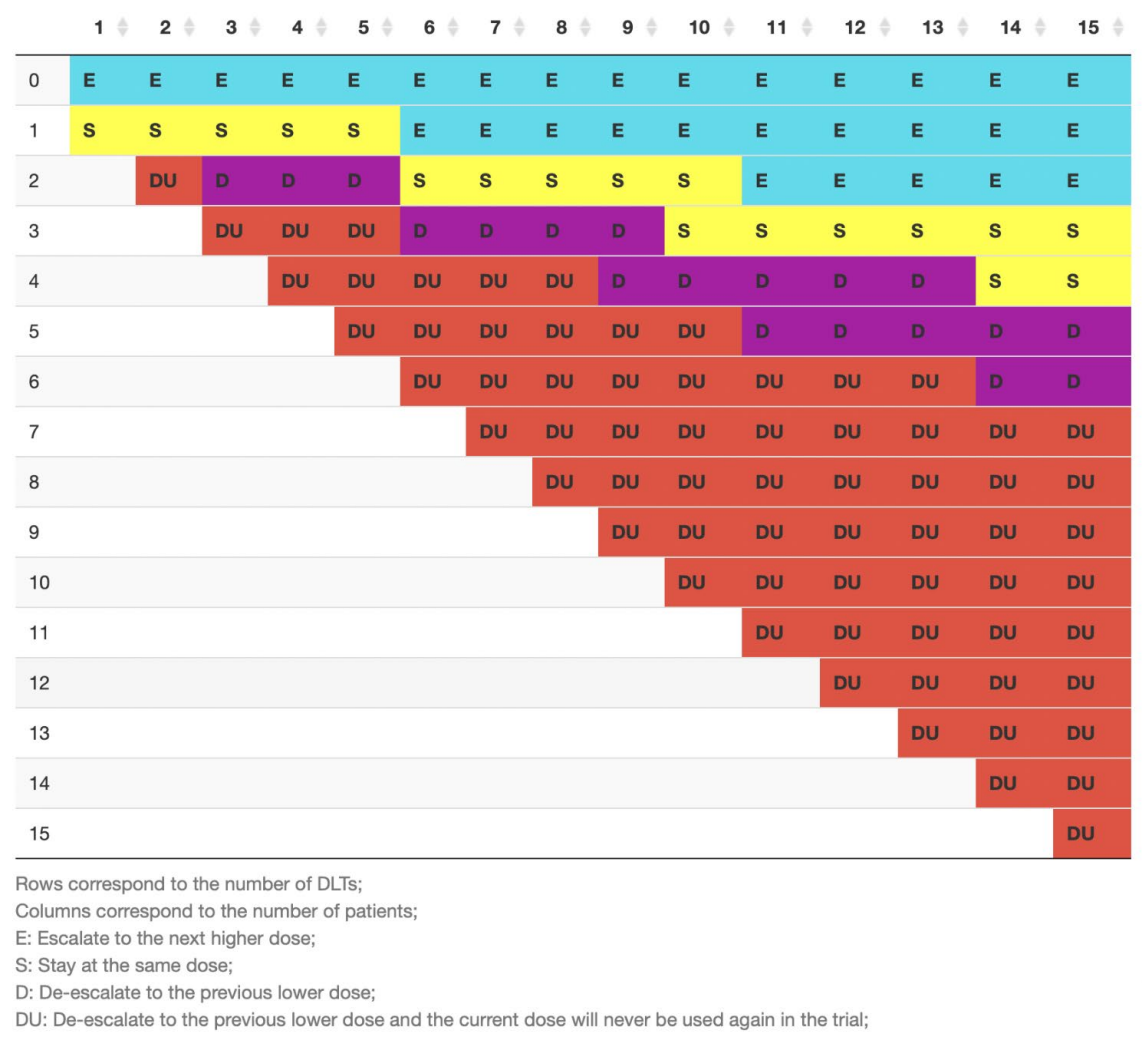
Simulation of the dose escalation according to the occurrence of adverse events. Unless an adverse event is registered, dose escalation until the target dose will follow with each patient

The study is designed as a single arm, multi-center dose escalation trial employing the i3 + 3 design assuming that 15 patients will be treated and an event rate of 20% is acceptable (Fig. 1) [16]. The study will treat 15 patients and escalate the dose up to a maximum level of ITV prescription dose-equivalent level of maximum 35.1 Gy to the 65–90% target encompassing isodose. With the assumption that 15 patients will be treated and a toxicity event rate of 20% ±5 is acceptable, a dose escalation schedule has been simulated [8].

The ITV dose will be escalated from 27.0 Gy to an ITV prescription dose-equivalent level of maximum 35.1 Gy while keeping the PTV prescription dose constant at 25 Gy after each patient if no dose limiting toxicities (DLT) have occurred (E). If a DLT is observed within the first five patients, the dose will not be further increased unless 5 patients were treated at the same dose (S) without occurrence of another DLT in one of these remaining four patients (Fig. 1). In case of a DLT in a second patient at the same dose level a dose de-escalation to the previous lower dose is employed and the current dose will never be used again in this trial (DU). If the ITV prescription dose-equivalent level of maximum 35.1 Gy is reached the remaining patients will be treated at this dose level, until the target cohort of n = 15 is reached. If no radiation associated adverse events occur, dose can be escalated with each recruited patient (i.e. row 0 and columns 1–5 in Fig. 1). The likelihood that dose escalation ensues too quickly with deleterious effects is very small.

### Patient population

Following inclusion criteria are required for participation:

- Patients with ischemic as well as nonischemic cardiomyopathies.
- Implanted ICD or CRT-D.
- Prior ≥ 1 failed CA (with endocardial ± epicardial approach based on the substrate location and the ECG morphology of clinical VT) procedure to control sustained MMVT using currently recommended mapping and ablation techniques.
- Patients in whom ablation is not feasible or contraindicated (e.g. LV thrombus or mechanical mitral and aortic valve).
- MMVT recurrence after CA on optimized antiarrhythmic and heart failure medication.
- Age ≥ 18 years.
- IRB-approved, written informed consent as documented by signature.

Exclusion Criteria [17]:

- Patients with MMVT who demonstrate:
  - Acute myocardial infarction.
  - Primary electrical disease (channelopathy).
  - Reversible and treatable cause (e.g., drug-induced or intoxication) of VT that can be adequately addressed otherwise.
  - A target that cannot be safely and precisely defined based on stereotactic radiotherapy accuracy requirements (e.g., anatomical interference from OARs, overlapping prior radiation therapy to the thoracic region).
  - Pregnancy or breastfeeding.
  - Inability to provide informed consent.

### Follow-up

After study intervention, patients will be followed up after one day, one week, and at 3, 6, 9, 12, 18 and 24 months or if any symptoms occur necessitating work-up. Study assessments at the different follow-up timepoints are shown in Table 1.

**Table 1:** Planned study assessments during follow-up

Follow-up	D1	W1	M3	M6	M9	M12	M18	M24
**Clinical examination**	X	X	X	X	X	X	X	X
**ECG**	X		X	X	X	X	X	X
**EP Map**	X							
**Lung function**	O			O				
**Chest/Cardiac-CT**	X		X			X		X
**FDG-PET**	O		O		O			O
**SPECT**	O		O		O			O
**MRI**	O		X		X			X
**Transthoracic echocardiography**	X		X		X			X
**ICD readouts**	X	O	X	X	X	X	X	X
**Medication use/dose**	X	X	X	X	X	X	X	X
**Blood samples**	X	O	O		O	O		O
**Chest X-ray**	O		O			O		
**CTCAE v5**	X		X	X	X	X	X	X
**PROMS**	X		X	X	X	X	X	X
**Physician reported QoL**	X		X	X	X	X	X	X

D = day; M = month; O = optional; PROMS = patient reported outcome measures; QoL = quality of life; X = mandatory; W = week. PROMS include EQ5D, SF-36, Treatment decision regret score

### Target definition and treatment delivery

All patients must undergo pre-procedural cardiac imaging (chest/cardiac CT).

Target volume delineation will be 4D-CT ITV based employing a 3–5 mm ITV to PTV margin. The ITV dose will be escalated from 27.0 Gy to an ITV prescription dose-equivalent level of maximum 35.1 Gy while keeping the PTV prescription dose constant at 25 Gy (Table 2). If feasible, software based electroanatomical mapping-to-radiotherapy planning registration will be employed [18]. The dose to the target (i.e. the VT substrate without PTV margin) will be increased by step-wise reduction of the prescribing isodose line (85% down to 65%).

Doses to OAR will be limited according to the recommendations published previously [19].

**Table 2:** Summary of dose and fractionation schedules (all doses in Gy)

Level	PTV (D95%≥)	ITV (D95%≥)	Dmax (D0.1 cc)	Isodose
1	25.0 Gy	27.0 Gy	28.5–30.0 Gy	~ 85%
2	25.0 Gy	29.7 Gy	31.5–33.0 Gy	~ 75%
3	25.0 Gy	32.4 Gy	34.5–36.0 Gy	~ 70%
4	25.0 Gy	35.1 Gy	37.5–39.0 Gy	~ 65%

### Outcome measures

The primary outcome of this trial is safety measured by registered radiation associated adverse events (AE) up to 90 days after study intervention. This includes any radiation associated serious adverse event (SAE; at least grade 4 or 5 according to CTCAE v5), in particular death, radiation pneumonitis requiring hospitalization, radiation pericarditis requiring hospitalization and decrease in LVEF ≥ 10% as assessed by echocardiography or cardiac MRI. The secondary outcome measures include efficacy endpoints (i.e. freedom from sustained VT/VF), non-radiation associated safety and patient reported outcome measures (PROMS) as shown in Table 3.

**Table 3:** Secondary outcome measures. QOL = quality of life

Efficacy	6-month survival free from electrical storm and incessant VT (binary endpoint) including an initial blanking period of 8 weeks
	Efficacy parameters will be assessed after an initial blanking period of 8 weeks, by comparing the 6 months prior to STAR with the 6 months after treatment, including the 8-week blanking period. All arrhythmic episodes occurring during the blanking period will be collected
	Sustained VT/VF burden as measured by number of post-treatment VT/VF episodes (ECG, ICD readouts). The main analysis will be performed by comparing the 6 months prior to STAR with the 6 months after treatment, which will include an 8-week blanking period
	Time to first sustained VT/VF recurrence as provided by ICD readouts, sustained VT/ICD shock and/or first VT storm
	Reduction of electrical storms and appropriate ATP and ICD shocks
	Need for antiarrhythmic drug use: type and dosages will be collected, and a drug index will be calculated
**Safety**	Overall survival and need for heart transplant or mechanical circulatory support: reported survival and heart transplant rates during follow-up
	Incidence of cardiac arrhythmic mortality or cardiac non arrhythmic mortality
	Non-radiation associated safety, e.g.: Hospitalization for acute heart failure necessitating inotropic therapy and if needed mechanical circulatory support except when other reason is apparent (e.g. acute coronary syndrome) ○ Cardiac tamponade ○ Major stroke or systemic embolism according to current diagnostic standards ○ ICD malfunction necessitating an interventional or operative approach
**Physician-reported and patient-reported QoL**	EQ5D, SF-35, QLQ-5, Treatment decision regret score
**Procedural technique outcome measures**	Error quantification of target definition and transfer from electrophysiology mapping to CT
	Correlation between dose to the target and VT number reduction
	Correlation between dose distribution to the target volume and scar formation
	Dose-volume constraints for OARs: as determined by organ-specific reported toxicity (CTCAE v5)
	The added value of additional imaging compared to standard CT imaging

### Statistical analysis

We will perform intention-to-treat (ITT) and per-protocol (PP) analysis of the primary dataset using multivariate and subgroup analysis to evaluate safety, efficacy, OAR and target doses, and treatment strategies, and pair this with CTCAE events, QoL, and PROMs, as appropriate. The primary (safety outcome), toxicity, will be considered in a time-to-event fashion and a complication-free interval and complication-free overall survival will be estimated using the Kaplan-Meier method. Mortality will be presented as a Kaplan-Meier analysis curve. In addition, toxicity will be summarized in a graph as cumulative frequencies of the standardized toxicity levels for each time point. Statistical analysis of effectiveness (secondary analysis) will be performed by comparing the number of VT events, ICD shocks, and ATP events between the six months prior to the six month after the intervention. Effectiveness is described by measures of location and scatter (95% confidence intervals) of the distributions of the features and differences to baseline. In general, confidence intervals will be derived from the score function for proportions and Hodges-Lehmann intervals for medians. The statistical significance level will be two-sided, α = 0.05.

Adverse events will be tabulated by organ or sub-organ system, intensity, and relatedness. During data analysis, relevant stratification criteria are applied to the datasets. Notably, several arrhythmogenic life-threatening entities will be included to determine whether STAR is a ubiquitous treatment or whether certain conditions are more suitable for this innovative approach. VT affects both women and men, irrespective of any stratification criteria.

Missing data and dropouts will be dealt with as independent right censoring. Missing data will be listed, showing reasons for unavailability. Sensitivity analyses of efficacy endpoints will be performed on the per-protocol analysis set defined as the subset of the ITT analysis set who have received protocol treatment and who have no other major protocol deviations thought to impact on the efficacy conclusions of the trial. Only patients who discontinued the study due to consent withdrawal will be replaced with new patients and should get only standard follow-up and no further study-specific follow up.

Statistical analyses will be performed using R version 4.0.2 (The R Foundation of Statistical Computing, Vienna, Austria).

### Trial organization and coordination

The DEFT-STAR study is an investigator-initiated trial designed by the principal investigators from the Radiation Oncology and Cardiology Departments of the University Hospital Zurich, Zurich, Switzerland. Additional study sites are currently being opened in the second stage of the trial. Participant recruitment, study intervention and follow-up are performed by physicians at the respective Radiation Oncology and Cardiology Departments. Data collection, data management, quality assurance and monitoring are performed by the Clinical Trials Unit, University of Zurich, Switzerland.

### Ethics and study participant safety

The study has been approved by the Cantonal Ethics Committee of Zurich, Switzerland (BASEC 2022 − 00262). This study will be conducted in compliance with the protocol, the current version of the Declaration of Helsinki, ICH-GCP, as well as all national legal and regulatory requirements. An independent data and safety monitoring board (DSMB) including a board-certified radiation oncologist and cardiologist/electrophysiologist has been established. The DSMC will review and evaluate the accumulated study data for subject safety, study conduct and progress, and make suggestions concerning the continuation, modification, or termination of the study. DSMB meetings will be scheduled before the trial start, after enrollment of the first 2 subjects and another 3 subjects, or at least every 6 months. The trial has been registered at clinicaltrials.gov (NCT05594368).

## Discussion

To our knowledge, DEFT STAR is currently the only ongoing dose-escalation trial in STAR. There are several ongoing randomized and non-randomized prospective clinical trials, a recent systematic search of clinicaltrials.gov revealed 16 ongoing or planned trials with a total planned participant count of 615 [20]. However, all of these reported a planned maximal radiation dose of 25 Gy (when reported).

A recent publication raised the hypothesis that the antiarrhythmic effect elucidated by radiotherapy might not actually be due to transmural fibrosis at radiation doses of 25 Gy [13]. In fact, the authors found in one explanted heart after treatment with STAR an increase in the expression of Na_v_1.2 and Cx43 – both integral for intermyocardial signal conduction. This may have led to an improvement in conduction velocity and could explain the narrowing of QRS complex in the patients treated with STAR in their study cohort. Improvement of conduction could in theory prevent unidirectional conduction block, which is a perquisite for MMVT due to reentry [12]. However, histopathologic and imaging evaluations of irradiated hearts suggests some degree of fibrosis and/or edema as compared to before STAR in a majority of publications which in turn can be proarrhythmogenic by increasing the heterogeneity of myocardial tissue [10, 15]. Moreover, a recently published animal study has convincingly demonstrated that a radiation dose of 40 Gy delivered in healthy or scarred myocardium leads to fibrosis, reduction in conduction velocity as well as reduced expression of Cx43 as early as 8 weeks after irradation [21]. CA is a well-established and studied intervention for the treatment of MMVT. The goal of CA indeed is scar homogenization (i.e. by inducing transmural fibrosis) eliminating critical isthmus for MMVT, making this the most reasonable endpoint for STAR.

The first patient was successfully treated in September 2022 without safety concerns. He experienced 3 appropriate ICD shocks due to fast monomorphic VT recurrences three months after STAR. Since then, he is free of sustained VA at last follow-up 6 months post STAR.

DEFT STAR is a phase II dose-escalation trial with a primary safety endpoint. Radiation-induced severe adverse events were infrequent, although systematic AE assessment and long-term reports are scarce [10, 22]. This trial aims to whether a dose-escalation for increased efficacy is safe in the investigated trial population.

## Data Availability

Not applicable.

## Abbreviations

ICM: Ischemic cardiomyopathy
NICM: Non-ischemic cardiomyopathy
MMVT: Monomorphic ventricular tachycardia
CA: Catheter ablation
OAR: Organs at risk
LVEF: Left ventricular ejection fraction
ATP: Antitachycardia pacing
STAR: Stereotactic arrhythmia radioablation
sVA: Sustained ventricular arrhythmia
VT/VF: Ventricular tachycardia/fibrillation
SCD: Sudden cardiac death
